# Prognostic and Diagnostic Significance of Annexin A1 in ypT3 Locally Advanced Rectal Cancer Patients

**DOI:** 10.3390/ijms27146512

**Published:** 2026-07-22

**Authors:** Diana Lavinia Pricope, Adriana Grigoraș, Gabriel Mihail Dimofte, Cornelia Amalinei

**Affiliations:** 1 Department of Morphofunctional Sciences I, Grigore T. Popa University of Medicine and Pharmacy Iasi, 700115 Iasi, Romania; dianalavinia64@gmail.com; 2 Department of Histopathology, Institute of Legal Medicine, 700455 Iasi, Romania; 3 Surgical Department, Grigore T. Popa University of Medicine and Pharmacy Iasi, 700115 Iasi, Romania; gdimofte@gmail.com; 4 2nd Clinic of Surgical Oncology, Regional Institute of Oncology, 700483 Iasi, Romania

**Keywords:** annexin 1, colorectal cancer, neoadjuvant therapy, rectal cancer diagnosis, rectal cancer prognosis

## Abstract

The prediction of the therapeutic response and of the patient outcome following neoadjuvant chemoradiotherapy (nCRT) in rectal cancer (RC) is still a challenging issue. The role of Annexin 1 (ANXA1) in the modulation of the tumor cells’ growth and metastasis has been recently demonstrated, being associated with aggressive pathological features and poor outcome in specific malignancies. The present study aimed to evaluate the association between ANXA1 expression, survival, and clinicopathological parameters of a group of patients diagnosed with ypT3 locally advanced rectal cancer (LARC) following nCRT. The study group consisted of 60 LARC patients with ypT3 tumor stage showing a tumor fragmentation pattern following nCRT. The clinicopathological characteristics and survival parameters in relation to ANXA1 immunohistochemistry characteristics and its scoring were evaluated. ANXA1 expression in tumor cells showed variable intense luminal membrane or combined luminal membrane and cytoplasmic location, excepting three negative cases. The statistical analysis demonstrated a significant association between high ANXA1 expression and ypN category (*p* < 0.001), perineural invasion (PnI) (*p* = 0.002), and lymphovascular invasion (LVI) (*p* = 0.021). Survival analysis showed that high ANXA1 expression is associated with a reduced overall survival (OS) (*p* = 0.001), while univariate Cox regression confirmed ANXA1 value as an independent predictor of prognosis (H = 5.922, *p* = 0.004). Our results support ANXA1 value as a potential biomarker of aggressive tumor behavior and poor prognosis in ypT3 LARC patients, with potential implications in diagnosis stratification, opening the prospective to apply precision oncology strategies. However, further studies are required to certify ANXA1 diagnostic and therapeutic relevance in RC patients.

## 1. Introduction

Although extensive research efforts aimed to elucidate colorectal carcinogenesis molecular mechanisms, colorectal cancer (CRC) is still considered a major global health challenge [1,2]. Worldwide reports in 2022 were that of 1,926,425 new CRC cases and 904,019 CRC-related deaths [2]. Moreover, assuming a relatively stable mortality rate, the epidemiological projections suggest a worrisome upward trend, considering that CRC incidence is expected to increase by around 22.5% by 2050, reaching approximately 2.36 million cases and about 1.11 million deaths [2].

Rectal cancer (RC) accounts for around one-third of all CRC cases, being associated with a global incidence of approximately 13.9 per 100,000 in men and 8.6 per 100,000 in women [3]. Among RC patients, locally advanced rectal cancer (LARC) is registered in approximately 5–10% of cases [4]. RC incidence has shown a steady annual increase between 2018 and 2022, of approximately 1%, now accounting for around 32% of all CRC, with about a 5% value increase by comparison with the middle of the first decade of the 21st century [5]. Another alarming trend is the recent increase in RC incidences among young people between 2013 and 2022, with an annual increase of 3% in people aged 20–49 years, compared to a modest increase of 0.4% in people aged 50–64 years in the same period of time [5].

Although neoadjuvant chemoradiotherapy (nCRT) leads to an improved survival rate, up to 30% of LARC patients may develop distant recurrences within five years, and pathological complete response rates are registered in only 15–27% of cases [3,6,7]. Moreover, age-standardized incidence and mortality rates are still disproportionately high in Eastern and Southern Europe compared to other geographical regions, regardless of the implementation of numerous screening programs in recent decades [2]. These disparities are probably associated with limited access to standardized adjuvant treatment and targeted therapies, as well as the burden of comorbidities, which can have a negative impact on patients’ outcome and survival [1,8].

Collectively, these data support the urgent need for continued and extended research efforts to understand the complex molecular interactions in the CRC microenvironment. Additionally, the insight into the CRC microenvironment is essential for the identification of novel biomarkers useful for early diagnosis and supports the development of more effective targeted therapeutic strategies for both colon and rectal cancer patients.

In this context, annexin A1 (ANXA1) has been recently identified as a promising new therapeutic target in different malignancies, including CRC [9]. The ANXA1 gene, located on human chromosome 9q12-q21.2, encodes the ANXA1 protein, a 38 kD molecule, mainly found in the cytosolic area of the plasma membrane [10]. Structurally, it contains a conserved C-terminal “core” domain, comprising four annexin repeats, forming a convex disk responsible for Ca^2+^ binding [10,11,12]. Furthermore, the ANXA1 protein contains a unique concave N-terminal “head” region, which plays a regulatory role [10,11,12].

Initially considered as an anti-phospholipase protein with anti-inflammatory properties, ANXA1 has lately been recognized as a “double-edged sword” cancer protein, exhibiting variable biological roles in several tumor types, including pancreatic, breast, gastric, thyroid, and colon and rectal cancers [13,14]. In support of this consideration, a reduced ANXA1 expression was associated with a poor clinical outcome in variable cancer types, including thyroid carcinoma and head and neck squamous cell carcinoma [15,16,17]. In contrast, other studies support the pivotal role of ANXA1 overexpression in colon and rectal carcinogenesis, as well as in tumor progression and metastasis, contributing to resistance to chemotherapy-based therapies and to a decreased overall survival rate [18,19,20,21,22].

ANXA1 achieves its effects via multiple signaling pathways, supporting cancer stem cell maintenance, along with the modulation of apoptotic pathways, tumor neoangiogenesis activation, and tumor immune evasion promotion [12,21,22]. It also fosters a highly immunosuppressive tumor microenvironment via the activation of M2 tumor-associated macrophages, the suppression of dendritic cells, and the impairment of CD8+ T-cell anti-tumor activity [12].

The current knowledge is that ANXA1 may be a potential biomarker that could be included in diagnostic and prognostic stratification panels in RC patients’ diagnosis, along with specific genetic, clinicopathological, and imaging data [7]. However, the available evidence regarding RC ANXA1 expression is still limited [20], and its role in ypT3 cases has not yet been studied.

Thus, based on these contradictory findings and considering the limited data available regarding ANXA1 expression in RC patients, this exploratory study aims to evaluate its expression in association with clinicopathological characteristics and survival outcome in a cohort of ypT3 LARC patients and to assess its potential value as a diagnostic and prognostic biomarker. Considering ypT3 tumor stage clinicopathological parameters’ low degree of heterogeneity, a more precise evaluation of the treatment response, along with an increased ANXA1 immunoexpression associated with residual disease, was expected, even though the study had its limitations.

## 2. Results

**Figure 1:**
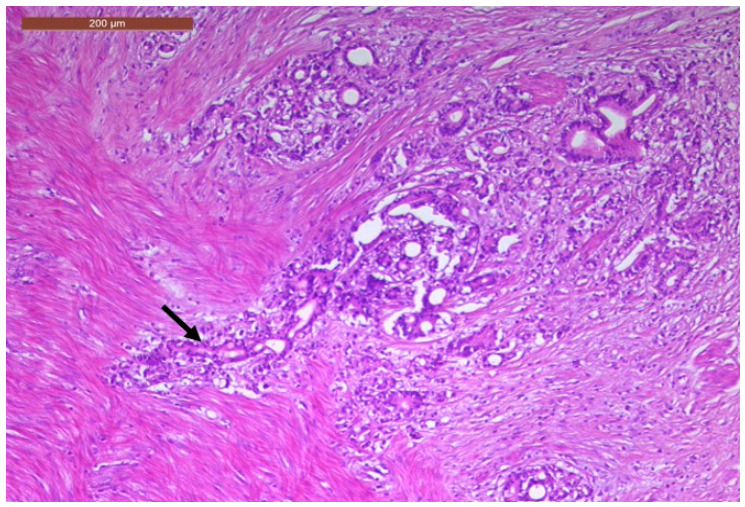
Tumor glands and small groups of tumor cells (arrow) infiltrating the eosinophilic smooth muscle cells of the muscularis propria (left side of the microphotograph) in an ypT3-stage LARC case (H&E staining, 100×).

**Figure 2:**
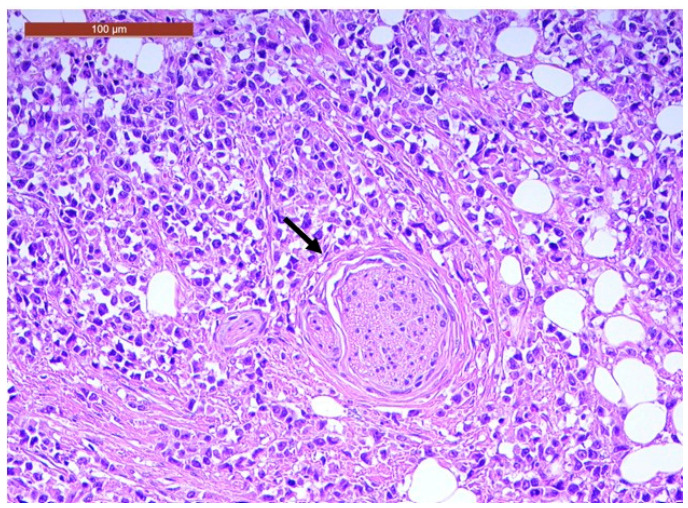
A ypT3-stage LARC case showing numerous basophilic tumor cells infiltrating a peripheral nerve (arrow) and the adipose tissue of the serosa (H&E staining, 200×).

**Figure 3:**
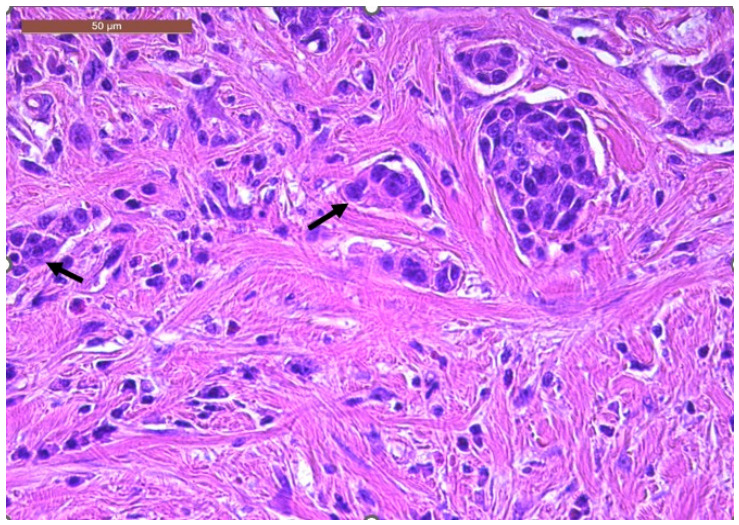
Small groups of cancer cells (arrows) disposed at the invasive tumor front in a ypT3-stage LARC case (PDC2), surrounded by an eosinophilic desmoplastic stroma (H&E staining, 400×).

**Figure 4:**
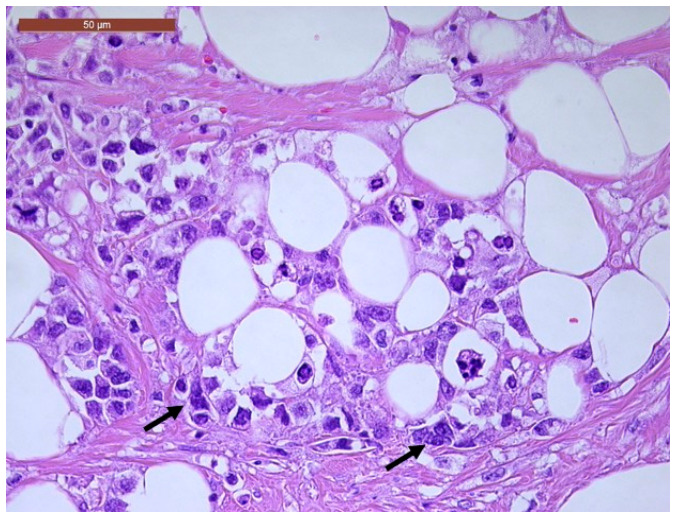
Individual or small clusters of cancer cells (arrows) infiltrating the white adipose cells in the invasive tumor front of a ypT3-stage LARC case (Bd2) (H&E staining, 400×).

The baseline data of the study group had been presented in a prior report [12]. Briefly, the study cohort mostly comprised men (n = 47; 78.3%) with a mean age of 63.93 ± 8.671 years. Most patients had cT3 clinical stage (n = 44; 73.3%) at presentation, with 90% of cases diagnosed as adenocarcinoma not otherwise specified (NOS). About half of cases had a favorable response to neoadjuvant chemoradiotherapy (ypN0) (n = 32; 53.3%). A significant number of cases showed lymphovascular invasion (LVI) (n = 29; 48.3%), EMVI (n = 39; 65%), IMVI (n = 29; 48.3%), and perineural invasion (PnI) (n = 40; 66.6%). BD2 was registered in only 7 cases (11.7%), PDC2 in 4 cases (6.7%) (Figure 1, Figure 2, Figure 3 and Figure 4), and 23 patients (38.4%) were recorded as non-survivors at their study enrollment [12].

### 2.1. Qualitative and Semi-Quantitative Analysis of ANXA1 Expression

**Figure 5:**
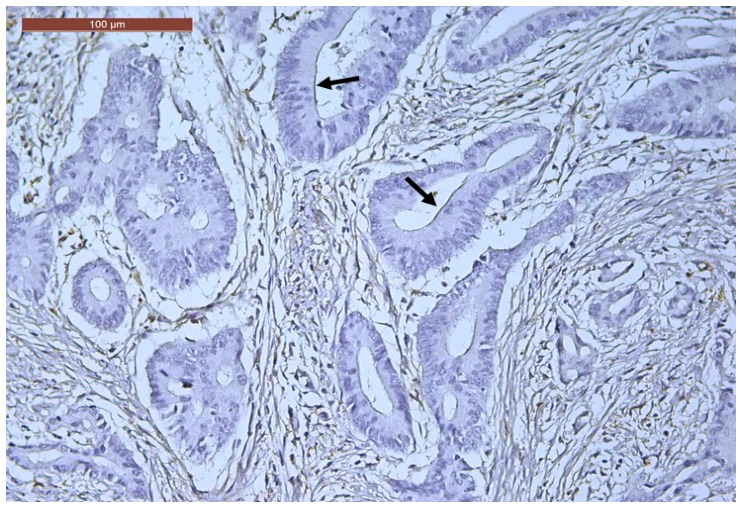
Weak luminal membrane ANXA1-positive immunoexpression in a few tumor glands (arrows) in a ypT3-stage LARC case (200×).

**Figure 6:**
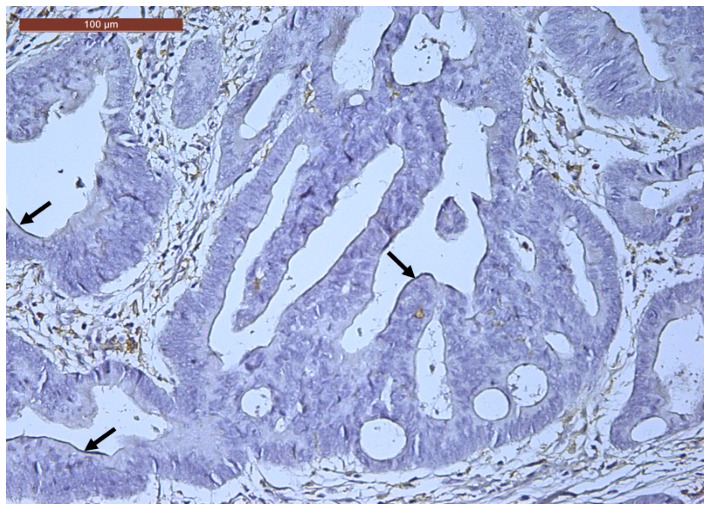
Focal moderately luminal membrane ANXA1-positive immunoexpression in tumor glands (arrows) of an ypT3-stage LARC case (200×).

**Figure 7:**
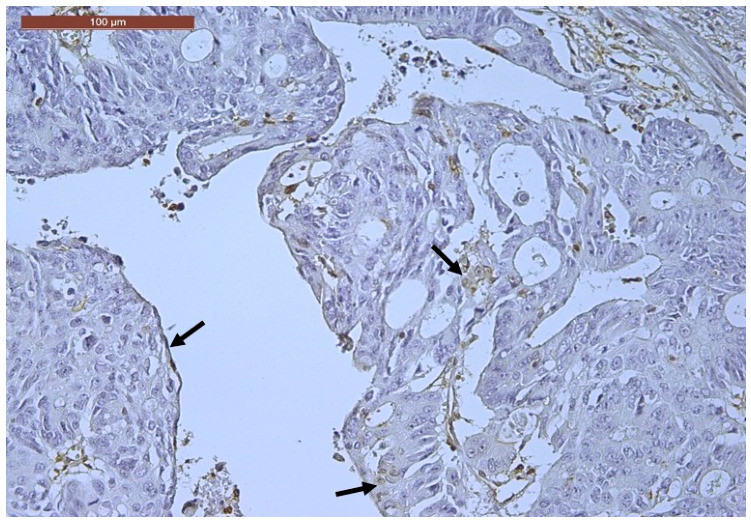
Focal moderately luminal membrane and cytoplasmic ANXA1-positive immunoexpression of tumor cells (arrows) in a ypT3-stage LARC case (200×).

**Figure 8:**
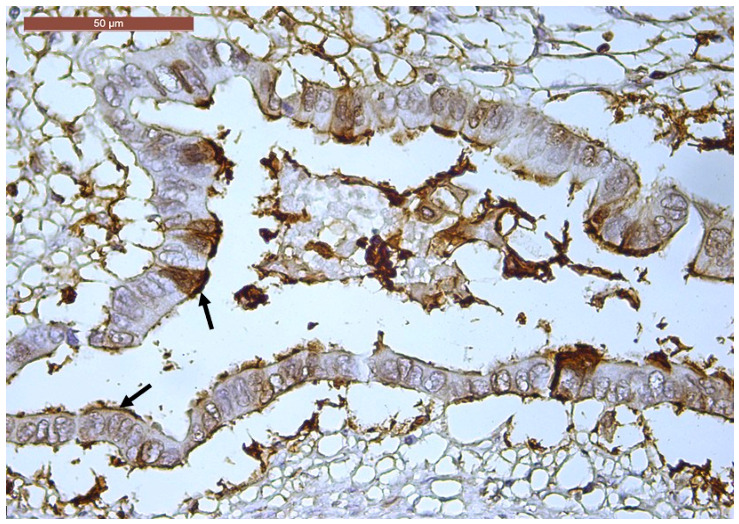
Strong luminal membrane and cytoplasmic ANXA1 expression in the gland (arrows) and intra-luminal tumor necrotic cells (in the middle of the tumor gland) in a ypT3-stage LARC case (400×).

ANXA1 tumor cells’ positive immunoexpression exhibited a well-defined luminal membrane location alongside a combined luminal membrane and cytoplasmic distribution. Most cases (n = 48; 80%) showed only a luminal membrane ANXA1-positive expression. Regarding the staining intensity, weak (1+) ANXA1 staining was observed in 22 cases (36.67%), whereas the other cases displayed moderate (2+) or strong (3+) ANXA1 staining (n = 35; 58.33%) (Figure 5, Figure 6, Figure 7 and Figure 8). A negative ANXA1 immunoexpression was observed in only three cases (5%). Based on the scoring system applied in the investigated tumors, ANXA1 expression was classified as low in 26 cases and high in 34 cases.

### 2.2. Relationship Between ANXA1 Immunoexpression and Clinicopathological Parameters

The cases exhibiting high ANXA1 expression showed a statistically significant association with ypN (*p* < 0.001), LVI (*p* = 0.021), and PnI (*p* = 0.002) in our study group (Table 1).

### 2.3. Relationship Between ANXA1 Immunoexpression and Survival Parameters

**Figure 9:**
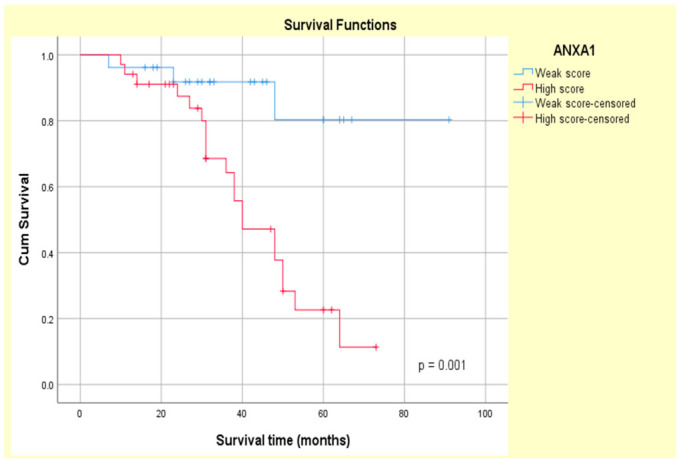
Kaplan–Meier overall survival curves stratified by ANAX1 expression in ypT3-stage LARC patients; x-axis represents the survival time, in months, while y-axis represents the survival probability; each of the curve downward steps corresponds to an event (death), while tick marks indicate censored observations; differences between groups were assessed using the log-rank test, with statistical significance defined as *p* < 0.05.

The survival analysis based on the ANXA1 score revealed a mean OS of 79.86 ± 6.05 months in patients with low ANXA1 tumor expression, whereas patients with high ANXA1 tumor expression had a significantly reduced mean OS (43.54 ± 3.42 months). Kaplan–Meier analysis further supported these findings, showing that high ANXA1 expression is associated with poor patient survival (log-rank Mantel–Cox, *p* = 0.001) (Figure 9). Moreover, the univariate Cox regression confirmed ANXA1’s prognostic significance in our study (H = 5.922, 95% CI: 1.746–20.093, *p* = 0.004).

## 3. Discussion

RC currently represents a major clinical challenge, with persistently rising morbidity and mortality, regardless of extensive research regarding its carcinogenesis molecular pathways, along with the demonstration of the tumor microenvironment’s involvement in the metastasis mechanism [20,23,24]. Moreover, patients with ypT3-stage exhibiting tumor fragmentation represent a clinically relevant subgroup characterized by uncertain prognosis and a suboptimal response to nCRT [23,25].

Although nCRT followed by total TME is the most accepted and recommended therapeutic management of LARC patients [26,27], the therapeutic response is heterogeneous, with only 15–20% of cases exhibiting a complete pathological response [28]. The pathological assessment of nCRT response includes the evaluation of tumor fragmentation, defined as the breakdown of the primary RC into multiple fragments of variable size and shape, a tumor pattern noticed in about 40% of LARC cases [24,29].

The ypT3-stage exhibiting tumor fragmentation shows microscopically residual tumor cells in the perirectal tissue beyond the muscularis propria layer, persistence of the initial pathological stage, and a dominant minimal to moderate response to therapy [23]. Therefore, the identification of novel diagnostic biomarkers is important, opening the prospective of the development of improved therapeutic strategies.

Among these diagnostic biomarkers, the first member of the annexin superfamily was described in the 1970s, its nomenclature being revised from renocortin to macrocortin, lipocortin-1, lipomodulin, and lately, ANXA1 [30]. Although it was initially identified as a putative anti-inflammatory factor, ANXA1 is highly expressed in inflammatory cells, such as monocytes, neutrophils, and eosinophils [30], showing a predominantly cytoplasmic location [31]. Subsequently, its expression has been documented across a broad range of cells or tissues, including cancer cells, showing a variable expression according to its involvement in variable processes, such as the regulation of cell survival, proliferation, and migration [12]. Moreover, ANXA1 modulates the macrophage activation in tumors and promotes an immunosuppressive tumor microenvironment by EGFR/STAT3 signaling axis regulation, contributing to cancer progression [16]. Additionally, ANXA1 has a pro-angiogenic role by the activation of the nuclear factor kappa B (NF-κB) signaling pathway in the tumor microenvironment [32]. ANXA1 also promotes tumor progression by the regulation of the PI3K/AKT pathway, a known oncogenic signaling cascade, enhancing tumor proliferation, invasion, and metastasis [33,34]. Furthermore, ANXA1 contributes to CRC 5-FU resistance by the regulation of the PKC/JNK/P-glycoprotein (P-gp) axis, leading to apoptosis inhibition and a decreased sensitivity to chemotherapy [12,35,36].

Although ANXA1 involvement in a wide range of malignancies has been widely recognized, its precise role in tumor growth and progression needs further clarification [37]. Moreover, ANXA1 heterogeneous expression across different cancer types leads to difficulties in understanding its function along with its significance. For instance, ANXA1 expression is upregulated in melanoma, pancreatic, lung, and gastric cancers, whereas its expression is downregulated in prostate, breast, and esophageal cancers [30,38]. These apparently opposite ANXA1 expression patterns in different malignancies probably represent the result of tissue-specific regulation by post-translational changes or the intervention of epigenetic factors, making the interpretation of its expression significance extremely difficult [30].

A reduced ANXA1 expression in colon and rectal tumor cells has been observed by comparison to other CRC niche components, according to a study performed on a small group of colorectal adenocarcinomas [39]. By contrast, another recent study on a large group of malignancies, comprising 1130 tumors, including CRCs, detected a dominant cytoplasmic and membrane ANXA1 expression in cancer tissues (53.3%) compared to a cytoplasmic, membrane, and nuclear expression in 165 normal tissues (57.0%) [40]. Complementary to this observation, another study has demonstrated a dominant tumor cell cytoplasmic ANXA1 expression in cases receiving preoperative chemotherapy [38].

Given these accumulated data, this study was designed to further investigate ANXA1’s potential diagnostic and prognostic biomarker value in LARC patients, considering the very limited amount of available information regarding this category of patients. The literature findings [38,40] are consistent with the results of our study in LARC patients, showing that ANXA1 positivity is predominantly expressed in the tumor cells’ luminal membrane or in both luminal membrane and cytoplasmic compartments of most cases, with only three tumors exhibiting a negative ANXA1 expression. Moreover, recent studies have shown that ANXA1 anti-apoptotic functions are mainly mediated by the NF-κB pathway activation, leading to the upregulation of pro-survival genes, such as *XIAP* and *BCL-2* [10,16]. In addition, ANXA1 plays a critical role in shaping an immunosuppressive CRC microenvironment by EGFR- and STAT3-dependent signaling cascade modulation [10,16]. Furthermore, membrane-associated ANXA1 expression promotes cancer cell migration, invasion, and metastatic dissemination via FPR2/ALX, EGFR-, MAPK/ERK-, and PI3K/AKT signaling axis activation [13,16].

Overall, these results support the hypothesis of ANXA1’s dual role as a regulator of apoptosis and immune response, activities associated with ANXA1’s cytoplasmic location, and as a regulator of tumor cell migration, a function associated with ANXA1’s dominant luminal membrane expression [41,42].

However, the variations in tumor stage, applied therapy, limited number of cases, and methodological approaches may partially result in conflicting findings regarding ANXA1’s immunoexpression of different cellular compartments and the discrepancies between different studies.

Only a limited number of studies investigated the potential prognostic role of ANXA1 in colon and rectal cancer and its association with variable clinicopathological parameters [19,20,38]. According to a recent study, no statistically significant associations could be demonstrated between ANXA1 overexpression and gender (*p* = 0.208), age (*p* = 0.68), or tumor grade (*p* = 0.86) in CRC patients [38], in agreement with the findings of our study. Similar findings were also reported by a previous study conducted on 172 RC patients following nCRT, without any statistically significant correlation between ANXA1 expression and patients’ gender (*p* = 0.344) or age (*p* = 0.117) [20]. Although another study performed on 210 CRC samples could not find an ANXA1 expression association with tumor differentiation, a significant positive association between its expression and gender was detected (*p* = 0.038) [19]. These contradictory findings may be attributed to the differences in the study design and the variable size of the analyzed cohorts. Moreover, the weak association between ANXA1 and tumor grade in CRC compared to other malignancies, such as breast cancer [43], may emphasize the tumor type variability and the tumor microenvironment’s complex characteristics.

While tumor grade reflects cellular proliferation and differentiation, lymph node metastasis, LVI, and PnI are more closely related to invasive and metastatic behavior involving cell adhesion, migration, and interactions with the tumor microenvironment. In this regard, most studies report an association between an increased ANXA1 expression and aggressive pathological features, supporting ANXA1 involvement in tumor progression [19,20,38]. In this context, a positive correlation between ANXA1 overexpression in CRC and lymph node metastases (*p* = 0.042), lymphatic invasion (*p* = 0.011), and venous invasion (*p* = 0.023) had been previously reported [19]. However, according to the same study, no significant association had been detected between tumor stage (*p* = 0.274), tumor location (*p* = 0.180), or tumor differentiation (*p* = 0.719) and ANXA1 overexpression [19]. Complementary data has also been added by another research team that reported a correlation between TRG (*p* = 0.009), post-therapy nodal status (*p* = 0.001), post-therapy tumor status (*p* < 0.001), or vascular invasion (*p* = 0.015) and ANXA1 expression but a negative correlation with PnI (*p* = 0.173) [20]. Moreover, another study in a cohort of CRC cases reported a positive correlation between an increased ANXA1 expression and lymph node metastasis (*p* = 0.001), as well as T stage (*p* = 0.011) and serosa invasion (*p* = 0.019) [38]. The same research group could not find any evidence of a correlation between tumor differentiation and ANXA1 increased immunoexpression and tumor size (*p* = 0.765) or tumor grade (0.086) [38]. These data show a partial agreement with the results of our study, with ANXA1’s high score being statistically associated with ypN, LVI, and PnI but not with TRG, tumor grade, or cT stage. The lack of correlation between ANXA1 expression and TRG or cT may be partially related to the relatively homogeneous distribution of these two parameters in our cohort, considering that most patients were classified as cT2 and showed TRG2–TRG3 responses following nCRT. Additionally, these findings should be interpreted in the context of our study group, which was restricted only to ypT3 LARC patients, limiting the parameters’ variability.

An association between ANXA1 expression and the survival outcome of CRC patients has been reported in several studies [19,22]. Our results also showed a significant association between ANXA1 high score and survival outcome in our study group (*p* = 0.001). Moreover, Cox regression analysis suggested ANXA1 value as an independent predictive factor for a reduced OS in ypT3 LARC patients (H = 5.922, *p* = 0.004). Consistent with our findings, another report noted a close association of ANXA1 expression with poor survival in CRC patients [22]. Furthermore, RC patients treated by nCRT followed by surgery, which exhibited a high ANXA1 tumor expression, had experienced a more aggressive clinical course, associated with reduced disease-specific survival (DSS) (*p* < 0.0001), metastasis-free survival (MeFS) (*p* = 0.0004), and local recurrent-free survival (LRFS) (*p* = 0.0001) [20]. Furthermore, Cox multivariate analysis confirmed ANXA1’s prognostic significance [20]. However, according to another study, the disease-specific survival was reduced in CRC patients with high ANXA1 tumor expression compared to patients with negative ANXA1 tumor expression, although this difference was not statistically significant (*p* = 0.6984) [19]. Taken together, these findings support that ANXA1 overexpression may represent a potential prognostic factor for poor survival in LARC patients. Moreover, ANXA1 could be considered a potential pathological diagnostic marker associated with tumor progression and metastasis.

Furthermore, ANXA1 may represent a potential therapeutic target in oncologic therapy and a topic of intensive research, including in the colon and rectal carcinogenesis pathways. In this context, punicalagin and granatin B, both pomegranate-derived compounds, have demonstrated anticancer effects in human CRC cell cultures and xenograft tumor models by the induction of apoptosis, cell cycle arrest, and the increase of the sensitivity to 5-FU, partially by ANXA1-related mechanisms [44,45]. In addition, MDX-124, a new humanized anti-ANXA1 antibody, has demonstrated in vitro and in vivo tumor growth inhibition by preventing ANXA1 interactions with formyl peptide receptors (FPR1/2) [14]. Furthermore, a phase Ib First-in-Human ATTAINMENT trial is currently evaluating in patients with advanced cancers the safety and the optimal dosage of MDX-124, in single use or in combination with standard oncologic therapies [46]. Initiated in 2023, dose-escalation cohorts up to 5 mg/kg have been completed without dose-limiting toxicity, while a 10 mg/kg dose cohort is currently ongoing [46]. Another preclinical study has also shown that MDX-124 reduces osteosarcoma cell migration and enhances therapy efficacy, supporting a planned phase Ib pediatric osteosarcoma trial that started in 2025 [47]. These clinical trials are opening new perspectives of tailored therapies in different cancers, including CRC.

Another therapeutic direction that may be applied in these oncologic patients is based on the observation that ANXA1 activity is associated with intestinal barrier dysfunction and disease severity in inflammatory bowel disease (IBD), a well-recognized CRC precursor [31,48]. Moreover, emerging evidence highlights the association between ANXA1 signaling and the gut microbiota dysbiosis [31,49] as another significant risk factor for both IBD and CRC [48]. In this context, Ac2–26 (an ANXA1-derived peptide) may restore the resolution pathways by enhancing IL-10 production via the reduction of pro-inflammatory cytokines, such as TNF-α and IL-1β, and by promoting the epithelial repair process in ulcerative colitis, suggesting its potential use in CRC [50]. Additionally, Ac2–26-loaded polymeric nanoparticles may improve epithelial healing and tissue regeneration in murine colitis by changing the gut microbiota, characterized by reduced *Escherichia–Shigella* and increased *Prevotellaceae* species [51]. However, the colon barriers in the delivery process are limiting the peptide- and nucleic acid-based oncotherapies [31,52]. Therefore, nanoparticle optimization, in addition to ANXA1-guided precision strategies, are required to apply these new discoveries in patients’ therapy and to provide increased therapeutic benefits [31,52].

Our study provides additional evidence regarding ANXA1 expression and its potential value as a prognostic factor of poor outcomes in ypT3 LARC, a subgroup of RC patients underexplored in this perspective. However, several study limitations should be considered. To begin with, due to its exploratory pattern, our study provides only a general view regarding ANXA1 expression in relation to the clinicopathological features and the survival parameters in a cohort of ypT3 LARC cases. Moving on, being conducted on a relatively small amount of cases, our preliminary statistical data should be cautiously interpreted and, as a consequence, further studies on large patient cohorts are required for validation through multiple comparative statistical analyses. Finally, the variability between the results of different studies may be attributed not only to the sample size but also to the different study designs and the variable interpretation of the immunohistochemical staining, emphasizing the requirement of a standardized approach to improve study reproducibility and results interpretability.

## 4. Material and Methods

### 4.1. Patients and Tissue Samples

The study group comprised 60 ypT3 LARC patients diagnosed in the Pathology Laboratory of the Regional Institute of Oncology Iasi during a seven-year period (2017–2023). This group was selected from a total of 238 LARC patients with variable ypT (ypT0–ypT4). Following neoadjuvant therapy, 133 tumors were classified as ypT3 stage. Among them, 73 tumors (54.89%) exhibited tumor shrinkage, while the other tumors (n = 60; 45.11%) showed a fragmented histological pattern. The inclusion and exclusion criteria have been previously reported [12], being illustrated in Table 2.

The tumor fragmentation following therapy was characterized by small clusters of tumor cells, separated by the main tumor mass by a minimum of 3 mm, lacking any continuity with it. The pathological tissue samples obtained by total mesorectal excision (TME) were diagnosed according to the last WHO classification [53].

Following neoadjuvant therapy, the tumor regression grade (TRG) was evaluated by the Dworak tumor response grading system. Accordingly, the tumors were classified as follows: complete response (TRG4), without any identifiable tumor cell due to total tumor regression; near-complete response (TRG3), with only few identifiable tumor cells included in a fibrotic area, accompanied or not by mucin; moderate response (TRG2), with marked fibrotic changes and rarely noticeable tumor cells or cell groups; minimal response (TRG1), with an evident tumor mass, associated with fibrosis, along with possible vasculopathy; and no response (TRG0) [54].

Tumor budding (Bd) and poorly differentiated clusters (PDCs) (Table 3), along with other main pathological features, have been reported for each case according to the current recommendations [53,55,56,57].

Overall survival (OS) was considered as the period of time from diagnosis to death or to the last follow-up, which was registered on 31 March 2023.

An integrated treatment strategy with nCRT followed by TME, the recommended standard care [26,27] at the time of their enrollment, was applied for LARC patients included in our study group. Therefore, all patients received long-course radiotherapy (45 Gy in 25 fractions plus a 5.4 Gy boost) with concurrent chemotherapy (CAP, CAPEOX, or FOLFOX). Surgery (Hartmann’s procedure, anterior resection, abdominoperineal resection, or TME) was performed following the neoadjuvant therapy at a 6–8-week interval.

The study complied with the ethical standards and was approved by the Ethics and Research Committee of Grigore T. Popa University of Medicine and Pharmacy, Iasi (no. 249/19 December 2022) and by the Ethics Committee of the Regional Institute of Oncology Iasi (no. 1267/4 July 2022).

### 4.2. Immunohistochemical Method

Paraffin-embedded tissue samples were sectioned at 4 μm, subsequently deparaffinized in xylene, and rehydrated in successive alcohol baths of decreasing concentrations (from 100% to 70%). The epitope unmasking was carried out using the HIER method, with a pH 8 solution, for 20 min (Vitro S.A. EDTA). The endogenous peroxidase activity was blocked using 200 μL of hydrogen peroxide 3% for 10 min. The incubation of the primary antibody, anti-ANXA-1 (mouse monoclonal antibody, clone 29, dilution 1/50, Vitro Master Diagnóstica, Sevilla, Spain), was achieved overnight at 4 °C. Signal development was performed using a compatible polymer detection system [Master Polymer Plus Detection System, acting as secondary antibody, with 3,3′-diaminobenzidine tetrahydrochloride (DAB) solution included] for 5–10 min at room temperature. Counterstaining was performed with Mayer’s hematoxylin, followed by dehydration and mounting. The external positive control was represented by tonsillar tissue, while the negative control was obtained by omission of the primary antibody.

ANXA1 immunoexpression was evaluated at 200× magnification by two pathologists with expertise (C.A. and G.A.), according to a previously published scoring system [20]. Inter-observer disagreements were reconciled through consensus. The ANXA1 score was calculated by a previously reported formula (Table 4). Tumors with a score above the median of all cases were classified as exhibiting a high ANXA1 expression [20].

### 4.3. Statistical Analysis

The statistical analysis was conducted using Microsoft Excel 2016 (Microsoft, Redmond, WA, USA) and SPSS version 25 (IBM, Armonk, NY, USA) programs. The association between clinical and pathological parameters and ANXA1 expression was evaluated using Pearson’s chi-squared test, while the survival analysis was conducted using the Kaplan–Meier method, with intergroup differences assessed by the log-rank (Mantel–Cox) test. Additionally, Cox proportional hazards regression analysis was used to assess the prognostic significance of ANXA 1 immunohistochemical expression. Statistical significance was achieved if *p*-value was <0.05.

## 5. Conclusions

Considering its dual role in the tumor microenvironment, ANXA1 is related to RC invasion and progression, being potentially associated with variable tumor cells’ luminal membrane and cytoplasmic expression.

Moreover, ANXA1 overexpression suggests its involvement in the development of an aggressive RC phenotype, supporting its potential value as a prognostic biomarker and a future therapeutic target in selected oncologic patients.

Although based on an exploratory study on a relatively reduced cohort size, ANXA1 expression significance may provide new insights into colon and rectal carcinogenesis. However, additional studies are required to confirm and validate its role within the complex RC microenvironment.

Additionally, interesting data may be obtained by future investigation of ANXA1’s signaling role in the regulation of the intestinal microbiota as a possible step in RC carcinogenesis.

## Figures and Tables

**Table 1:** Relationship between ANXA1 expression and clinicopathological features of the study group.

Clinicopathological Characteristics	ANXA1 Immunoexpression		# *p*-Value
	Low (n; %)	High (n; %)	
**Age**			*p* = 0.993
45–55 years	5 (8.3%)	7 (11.7%)	
56–65 years	9 (15%)	12 (20%)	
66–75 years	10 (16.7%)	13 (21.7%)	
76–85 years	2 (3.3%)	2 (3.3%)	
**Gender**			*p* = 0.817
Women	6 (10%)	7 (11.7%)	
Men	20 (33.3%)	27 (45%)	
**Clinical T stage**			*p* = 0.052
T2	-	3 (5%)	
T3	23 (38.3%)	21 (35%)	
T4	3 (5%)	10 (16.7%)	
**Clinical N stage**			*p* = 0.378
N0	1 (1.6%)	1 (1.6%)	
N1	5 (8.3%)	2 (3.3%)	
N2	16 (26.7%)	27 (45%)	
Nx	4 (6.7%)	4 (6.7%)	
**ypN category**			*p* < 0.001 *
yN0	16 (26.7%)	16 (26.7%)	
yN1	9 (15%)	12 (20%)	
yN2	1 (1.6%)	6 (10%)	
**Histological type**			*p* = 0.602
AC NOS	24 (40%)	30 (50%)	
mucinous AC	2 (3.3%)	4 (6.7%)	
**Grading**			*p* = 0.302
low grade	22 (36.7%)	25 (41.6%)	
high grade	4 (6.7%)	9 (15%)	
**TRG**			*p* = 0.869
TRG 0	1 (1.6%)	1 (1.6%)	
TRG 1	10 (16.7%)	15 (25%)	
TRG 2	12 (20%)	16 (26.7%)	
TRG 3	3 (5%)	2 (3.3%)	
**LVI**			*p* = 0.021 *
Negative	17 (28.3%)	12 (20%)	
Positive	9 (15%)	22 (36.7%)	
**IMVI**			*p* = 0.073
Negative	16 (26.7%)	13 (21.6%)	
Positive	10 (16.7%)	21 (35%)	
**EMVI**			*p* = 0.251
Negative	19 (31.7%)	20 (33.3%)	
Positive	7 (11.7%)	14 (23.3%)	
**PnI**			*p* = 0.002 *
Negative	23 (38.4%)	17 (28.3%)	
Positive	3 (5%)	17 (28.3%)	
**Bd**			*p* = 0.161
Bd1	22 (36.6%)	27 (45%)	
Bd2	4 (6.7%)	3 (5%)	
Bd3	-	4 (6.7%)	
**PDC**			*p* = 0.657
PDC1	24 (40%)	31 (51.7%)	
PDC2	2 (3.3%)	2 (3.3%)	
PDC3	-	1 (1.6%)	

# Pearson’s χ^2^ test—significant *p*-value < 0.05 (*). AC—adenocarcinoma; Bd—tumor budding; EMVI—extramural vascular invasion, IMVI—intramural vascular invasion, LVI—lymphovascular invasion; N—regional lymph nodes; NOS—not otherwise specified; N0—no regional lymph nodes involvement; N1—1–3 regional lymph nodes involvement; N2—4–6 regional nodes involvement; Nx—lymph nodes cannot be assessed; PDC—poorly differentiated clusters; PnI—perineural invasion; T—tumor; TRG—tumor regression grade; ypN—lymph node status following neoadjuvant therapy.

**Table 2:** Inclusion and exclusion criteria for cases selection.

Inclusion Criteria	Exclusion Criteria
>18 years of age	<18 years of age
previously neoadjuvant therapy administration * followed by TME	surgical resection without prior neoadjuvant therapy
	completed neoadjuvant therapy with withheld surgery owing to patient refusal
	‘watch-and-wait’ strategy
ypT3-stage diagnosis	ypT stages other than ypT3
tumor fragmentation as a histological response following therapy	tumor shrinkage pattern as a histological response following therapy

* radiotherapy combined with concurrent chemotherapy with capecitabine, FOLFOX (leucovorin calcium, fluorouracil and oxaliplatin), or CAPEOX (capecitabine and oxaliplatin); TME—total mesorectal excision.

**Table 3:** Tumor budding and poorly differentiated cluster evaluation system [55,56].

**Bd**	**Bd Count**	**Bd Score**
single tumor cells or small clusters (≤4 cells) at ITF	0–4 cells	Bd1
	5–9 cells	Bd2
	≥10 cells	Bd3
**PDCs**	**PDC Count**	**PDC Score**
clusters of ≥5 tumor cells at ITF	0–4	PDC1
	5–9	PDC2
	≥10	PDC3

Bd—tumor budding; ITF—invasive tumor front; PDC—poorly differentiated cluster.

**Table 4:** ANXA1 expression scoring system.

Staining Intensity in Tumor Mass	Staining Intensity Score	Percentage of Stained Cancer Cells	Final Score Formula	Category
0–25%	1	0–100%	Σ*Pi*(*i* + 1)	Low expression (≤125)
25–50%	2			
>50%	3			High expression (>125)

*i*—staining intensity; *Pi*—the percentage of stained cancer cells.

## Data Availability

The original contributions presented in this study are included in the article. Further inquiries can be directed to the corresponding authors.
